# Preparation, Application and Enhancement Dyeing Properties of ZnO Nanoparticles in Silk Fabrics Dyed with Natural Dyes

**DOI:** 10.3390/nano12223953

**Published:** 2022-11-09

**Authors:** Haijuan Du, Mengyuan Yue, Xin Huang, Gaigai Duan, Zhihui Yang, Weihan Huang, Wenjie Shen, Xiangfeng Yin

**Affiliations:** 1 College of Textiles, Zhongyuan University of Technology, Zhengzhou 450007, China; 2 Jiangsu Co-Innovation Center of Efficient Processing and Utilization of Forest Resources, International Innovation Center for Forest Chemicals and Materials, College of Materials Science and Engineering, Nanjing Forestry University, Nanjing 210037, China

**Keywords:** ZnO nanoparticles, morphology, dyeing properties, fastness, SEM and TEM images, FTIR spectroscopy

## Abstract

In this study, ZnO nanoparticles were prepared by a hydrothermal method with varying the reaction times, material ratios and reaction temperatures. The samples were characterized by scanning electron microscopy (SEM), transmission electron microscopy (TEM), X-ray Diffraction (XRD) and Fourier infrared spectroscopy (FTIR). It was shown that the material ratio significantly affected the structure and morphology of the synthesized ZnO nanoparticles, and then the uneven nano-octahedral structure, uniform nano-octahedral structure, nano-tubular structure, and nano-sheet structure could be obtained successively. The synthesized ZnO nanoparticles as mordant were used for the dyeing of silk fabrics with different natural dyes (tea polyphenols and hematoxylin). Moreover, they could improve the dyeing properties and fastness (wash and light) on silk fabrics to a certain extent.

## 1. Introduction

With the rapid development of nanomaterials, the research and development of nanomaterials has attracted extensive attention from researchers all over the world [1,2,3,4,5]. Nano zinc oxide is favored for its unique physical and chemical properties worldwide [6]. Nano zinc oxide has many advantages, including strong catalytic activity, a small size, a large specific surface area, a lowered environmental impact, and being reusable [7]. Moreover, compared with other metal oxide nanoparticles, ZnO nanoparticles are easy to prepare, as the main advantages of nano zinc oxide are that it is a biocompatible and low-cost material [8,9]. In recent years, nano zinc oxide has shown potential for applications in biopharmaceuticals, optoelectronic devices, ceramic processing, rubber products, textile printing and dyeing, sewage treatment and other industries [10,11,12,13,14]. Over the years, the preparation of nanoparticles has been described using several methods, such as hydrothermal [15,16], microwave-assisted [17,18], sol–gel [19,20], self-assembling [21], and chemical precipitation [22,23,24,25]. Based on the advantages of good uniformity of microcrystalline size and significant cost efficiency of industrial production, the hydrothermal method is more preferred by researchers [26,27]. Moreover, nanotechnology can help control particle size to fit functions that can find more applications for its use. For example, nanoparticles are considered excellent candidates as drug-delivery vehicles. In addition, ZnO nanoparticles have also been reported to bring an improvement in the mechanical and antimicrobial property of silk fibers, which can be used as potent surgical suture material [28].

In recent years, with improvements in living standards, green and safe back-to-nature ways have become a pursuit for many people. A renewed international interest has arisen in natural dyes as they are among the promising options for developing a greener textile dyeing process [29,30]. Natural plant dyes applied to textile dyeing have been reported to confer a variety of health benefits and functionalities on textiles, such as UV protection, antibacterial activity, deodorization, anti-carcinogenicity, and anti-oxygenation [31,32,33,34]. However, due to the low affinity between the colorants and the fibers when natural dyes are used in textile dyeing, they cannot be well fixed on the fibers, resulting in poor color fastness of the dyed fabrics [35,36,37]. Metal salt as mordant in the traditional color fixing process can improve the color fastness of dyed fabrics, but the use of metal salt pollutes water quality and the environment, and the color of dyed fabrics changes greatly. Moreover, a greater number of metal ions on the fabric can cause partial damage to the human body, such as skin allergy and itching [38]. In contrast, reports regarding any negative effects of nano zinc oxide on the dyeing properties and fastness of natural-dye dyed fabrics are rare.

We prepared nano-ZnO with different morphologies by a hydrothermal method. The prepared nano ZnO nanoparticles as mordant have been used to investigate the impact of the dyeing properties and fastness (wash and light) on silk fabric dyeing with natural dyes (tea polyphenols and hematoxylin). Overall, the introduction of new nanomaterials into traditional dyeing and finishing processes was a small innovation. The following focus is to figure out the mechanism of nanomaterials in functional textiles in order to better achieve the desired results [39,40].

## 2. Materials and Methods

### 2.1. Materials and Methods

Sappanwood and tea-leaves were purchased from Shangyuantang Pharmacy in Nanjing City, Jiangsu Province, China. Double crepe silk fabric was purchased from WENSLI, Hangzhou, China. All chemical reactants (zinc acetate, sodium hydroxide and ethanol) were purchased from Sinopharm (Beijing, China) and used without any further purification steps. Absorbance spectra of the staining solution before and after staining were performed using an Agilent CARY 5000 Scan UV-Vis-NIR spectrophotometer (Germany). Morphological characteristics of the ZnO nanomaterials were observed by field emission scanning electron microscopy (Hitachi Regulus 8100, Japan) and transmission electron microscopy (TEM, FEI TALOS F200X 200 kV, Hillsboro, OR, USA). The compositional quality of the synthesized materials was characterized by Fourier transform infrared spectroscopy (TENSOR37, China) in the mid-infrared (400 to 4000 cm^−1^) range. Color strength (K/S) of the dyed samples was tested by a Datacolor measuring and matching instrument (SF600X, China). A sunlight fastness test of the dyed fabrics was performed using a sunlight fastness meter (Q-SUN-B02, China). A soaping test of the dyed fabrics was performed using a soaping machine (SW-24E, China). The crystal structure of the samples was analyzed by X-ray diffraction in angle range (10–80°) with a diffractometer (Rigaku Smartlab).

### 2.2. Colorant Extraction

#### 2.2.1. Hematoxylin

To extract the hematoxylin colorant, sappanwood roots were first washed and dried and then chopped and powdered. Then, we selected fermentation times (48 h), fermentation solvents and extraction solvents (ethanol:water (3:7, *v*/*v*)) to extract plant dyestuff. The extracting solution was filtered and concentrated in a rotary evaporator flask to 1/3 of the original volume. The concentrated dye solution was freeze-dried under low temperature to obtain hematoxylin.

#### 2.2.2. Tea Polyphenols

**Figure 1:**
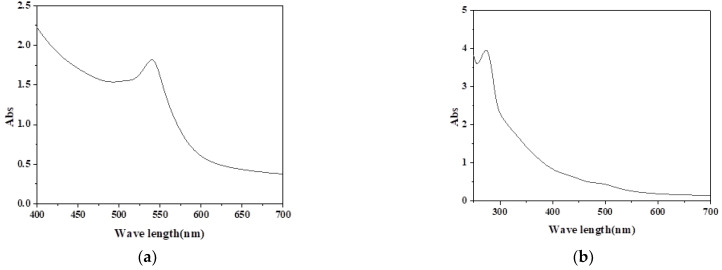
The absorbance spectra of hematoxylin (**a**) and tea polyphenols (**b**).

Powdered tea was soaked in a 2 L mixture solution of alcohol and water in a beaker at a volume ratio of 3:7 for 100 g. The excess alcohol and water were evaporated in a rotary evaporator at 75 °C. The solution was collected in a rotary evaporator flask and dried in a spray dryer at 170 °C for powder production. The dyed solution exhibited the absorbance spectra of the two colorants as shown in Figure 1.

### 2.3. Preparation

For hydrothermal growth, a 0.1 mol per liter of zinc acetate (Zn(CH_3_COO)_2_·2H_2_O) and 1 mol per liter of sodium hydroxide (NaOH) combined solution was prepared in volumetric flasks to be tested at different hydrothermal reaction times (6 h, 12 h), material ratios of Zn^2+^ substance and OH^−^ substance (Zn^2+^:OH^−^ (2:1, mol/mol), Zn^2+^:OH^−^ (1:1, mol/mol), Zn^2+^:OH^−^ (1:2, mol/mol), Zn^2+^:OH^−^ (1:4, mol/mol)), and hydrothermal reaction temperatures (100 °C, 130 °C). The preparation conditions for different samples of ZnO nanoparticles are shown in (Table 1).

The reaction was placed on a thermostatic magnetic stirrer at a temperature of 50 °C and a speed of 500 r/min. The sodium hydroxide solution was added dropwise to the zinc acetate solution, and the reaction lasted for 30 min. The mixed reacted solutions were transferred to the hydrothermal reactor, which was heated at the set temperature for 0.5 h and then cooled to room temperature at a rate of 10 °C·h^−1^. After the hydrothermal reaction, the resulting product was filtered and rinsed thoroughly with deionized water to remove the excess reactants from its surface. Finally, the samples were dried in an oven at a constant temperature of 60 °C.

### 2.4. Mordanting and Dyeing

The dyeing process was a simple water bath dyeing under continuous agitation with an alcohol ratio of 1:50, and dye concentration (2% on weight the fabric). The dyeing temperature was 80 °C with a holding time of 60 min, which started from 40 °C with an increase in velocity of 2 °C/min. Holding at 80 °C for 30 min, 0.1 g/L mordant was added and the fabrics continued to be dyed at 80 °C for 30 min. After the dyeing process was completed, the fabrics were washed under running water and dried in a cool place at room temperature.

### 2.5. Color Measurements

Dyed samples were analyzed by measuring the reflectance curve between 350 and 750 nm with the spectrophotometer with illuminant D_65_ at 10 degree observer. For each sample, three measurements at three different sections were made, and the average values were reported. Color strengths (*K*/*S*) of dyed samples were calculated using the Kubelka-Munk equation:

*K*/*S* = (1 − *R*)^2^/2*R* (1)

where *R* is the observed reflectance, *K* is the absorption coefficient, and *S* is the light scattering coefficient. In general, the greater the *K*/*S* value, the deeper the color on the fabric [34,35].

### 2.6. Determination of Dye Exhaustion

Dye uptake was determined by measuring the absorbance (at the wavelength of maximum absorbance, λ_max_ ≈ 540 nm) of the dye bath solution before and after dyeing. According to the Beer–Lambert law, when monochromatic light passes through the solution, the absorbance *D* is proportional to the concentration *C* of the dye in the solution, so there is

(2) $$\%\;Dye\;exhaustion\;=\frac{C_{0}-C}{C_{0}}\times 100=\frac{mD_{0}-nD}{mD_{0}}\times 100$$

where, *C*_0_ is the concentration of the dye in the original dye solution, *C* is the concentration of the dye in the dyeing residue, *D*_0_ is the absorbance of the original dye solution diluted m times, *D* is the absorbance of the dyeing residue diluted *n* times, m is the dilution factor of the original dye solution when measuring the absorbance, and *n* is the dilution factor of the dyeing residue when measuring the absorbance [31].

### 2.7. Fastness Properties

The wash and light fastness of dyed fabrics were tested in the light of ISO 105-C01, ISO 105-B02, respectively.

## 3. Results

### 3.1. Structure and Morphology

**Figure 2:**
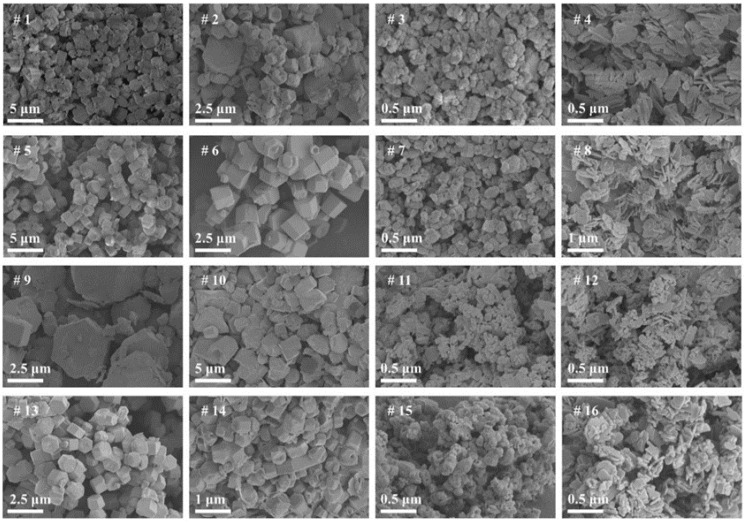
SEM images of nano zinc oxide prepared under different reaction conditions (with different reaction temperatures, reaction times and material ratios).

The different morphologies of ZnO nanoparticles were prepared by the hydrothermal method under different reaction conditions (reaction temperature, reaction time, material ratio), and measured by scanning electron microscopy (SEM) as shown in Figure 2. The SEM results of Figure 2 (^#^5–^#^8) show that when the hydrothermal reaction temperature is 130 °C and the reaction time is 6 h, the Zn^2+^:OH^−^ material ratio changes (2:1, 1:1, 1:2, 1:4), and then under these conditions, the uneven nano-octahedral structure, uniform nano-octahedral structure, nano-tubular structure, and nano-sheet structure can be obtained successively. This feature indicates that the morphology of ZnO nanoparticles gradually becomes homogeneous as the OH^−^ concentration increases. With a further increase in OH^−^ concentration, the surface of ZnO nanoparticles is etched on the original octahedral structure, and the central position of ZnO particles is etched to form a nano-tubular structure. When the Zn^2+^:OH^−^ material ratio is 1:4, the deposition of zinc oxide is significant due to the high concentration of OH^−^, and the longitudinal growth of nano-ZnO crystals is inhibited and a nano-sheet structure is formed.

The SEM images in Figure 2 (^#^1–^#^4 and ^#^5–^#^8) show the morphology of ZnO nanoparticles grown at different hydrothermal temperatures (100 °C and 130 °C). It can be seen that the surfaces of nano-ZnO crystals grown at 100 °C are rough and irregular, while the surfaces of nano-ZnO crystals grown at 130 °C are smooth and regular.

The SEM images in Figure 2 (^#^5–^#^8 and ^#^13–^#^16) are the morphology of nano-ZnO crystals grown at different hydrothermal times (6 h and 12 h). With the increase in hydrothermal time, the morphology of nano-ZnO crystals becomes irregular and the nano-surface becomes rough.

The SEM images in Figure 2 (^#^9–^#^12 and ^#^13–^#^16) show the morphology of nano-ZnO crystals grown at different hydrothermal temperatures (100 °C and 130 °C). With the increase in hydrothermal temperature, the size of nanoparticles further increases and the nano-surface becomes smooth.

**Figure 3:**
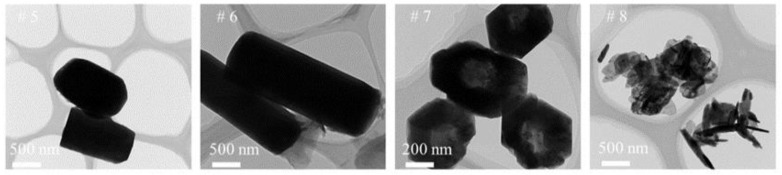
TEM images of nano zinc oxide prepared under different reaction conditions (^#^5–^#^8 with different material ratios).

Further studies on the morphology and structure of the as-synthesized ZnO particles were investigated by TEM (Figure 3). Samples (^#^5–^#^8) were selected to test which ones agreed with the SEM results. Moreover, it could be observed that the diameter of the ZnO particles was approximately 500–1500 nm.

### 3.2. X-ray Diffraction (XRD) and FTIR Analysis for the ZnO Nanoparticles

**Figure 4:**
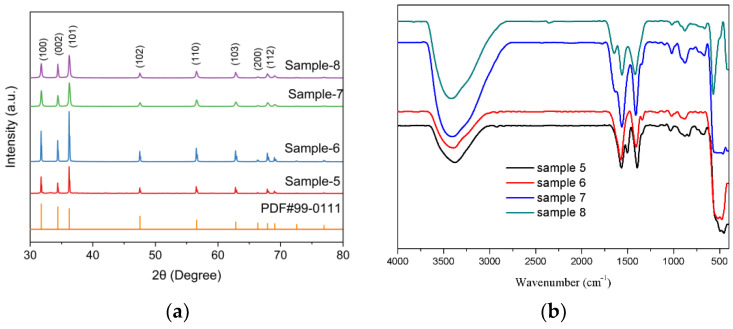
The XRD patterns (**a**) and FTIR spectra (**b**) for ZnO-NPs.

Figure 4a showed the observed XRD patterns of the synthesized ZnO nanoparticles with identified obtained peaks such as (100), (002), (101), (102), (110), (103), (200) and (112) are related to the hexagonal (wurtzite) structure of ZnO. The corresponding XRD peaks of samples 5 and 6 were stronger and sharper, indicating that the samples crystallized well and the average size of particles was bigger than 7and 8 [41,42].

Figure 4b showed the FTIR spectrum of samples 5–8, which was acquired in the range of 400–4000 cm^−1,^ The different concentrations of adsorbed water molecules and hydroxyl groups in the sample can be demonstrated by the band strength corresponding to the tensile vibration of the O-H group (3000–3620 cm^−1^). The stretching modes of vibrations in asymmetric and symmetric C=O bonds are observed at 1560 and 1410 cm^−1^, respectively. The band at 450 cm^−1^ is correlated to ZnO [15].

### 3.3. Color Strength of Dyed Fabric with Hematoxylin and Tea Polyphenols

**Figure 5:**
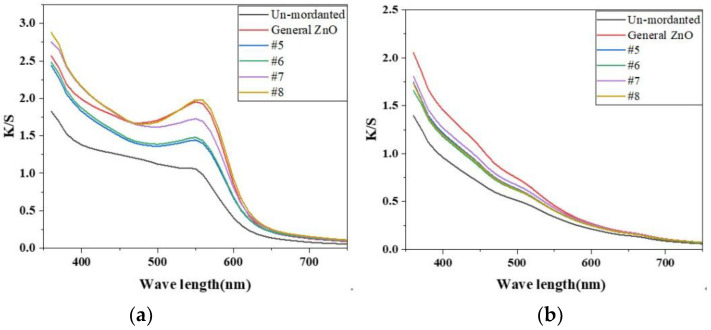
The K/S of the silk fabric dyed with hematoxylin (**a**) and tea polyphenols (**b**) under the action of different morphology nano-zinc oxide mordants at wavelengths of 350–750 nm.

Figure 5 shows the color strength (K/S) of the silk fabrics dyed with hematoxylin and tea polyphenols under the action of different morphology nano-zinc oxide mordants at wavelengths of 350–750 nm. Compared with the unmordanted fabric, the color of the mordant-treated samples became more intense, resulting in greater K/S values. It can be clearly seen that the color strength of nano-ZnO is more intense and the K/S value is greater than that of the original ZnO sample fabric dyed with hematoxylin, and the original color of the dye on the fabric can be well maintained.

### 3.4. Dye Exhaustion

**Figure 6:**
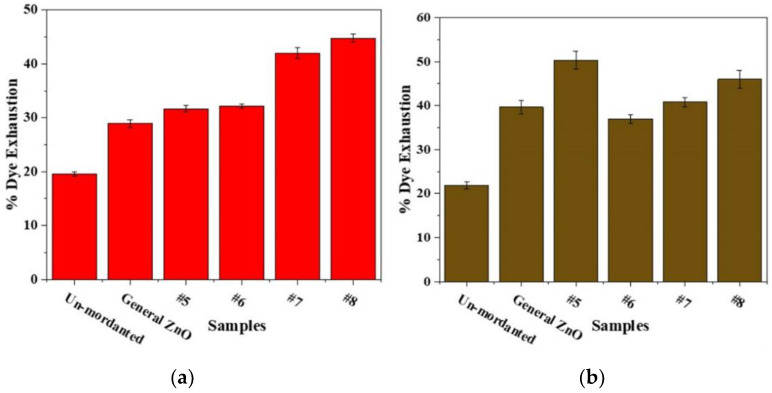
The rates of dye exhaustion of hematoxylin (**a**) and tea polyphenols (**b**) on silk fabric under the action of different morphology nano-zinc oxide mordants.

The rates of dye exhaustion of hematoxylin and tea polyphenols on silk fabric under the action of different morphology nano-zinc oxide mordants are shown in Figure 6. The addition of mordant greatly increased the dye exhaustion. It was found that the nano-zinc oxide mordants were more helpful in increasing dye exhaustion, followed by ordinary zinc oxide and unmordanted silk fabrics. Compared with ordinary zinc oxide, the rates of dye exhaustion of dyed fabrics with nano zinc oxide are obviously improved. Through the SEM images of nano zinc oxide, it can be concluded that the different rates of dye exhaustion were caused by the morphology of nano zinc oxide, which may be due to the surface area increase effect of the synthesized ZnO nanoparticles. In addition, the rates of dye exhaustion of hematoxylin and tea polyphenols are very different for the more hydroxyl groups of tea polyphenols’ structure anchoring onto ZnO nanoparticles.

### 3.5. Fastness Properties

The fastness (wash and light) of the silk fabrics dyed with hematoxylin under different mordants are shown in (Table 2). It was found that all the dyed fabrics had poor wash and light fastness. Because the molecular structure of hematoxylin dye itself has multiple hydroxyl groups, it has good water solubility, thus leading to the decrease in wash fastness. Under the light condition, the conjugated system of dye itself is damaged by photo-oxidation and fades. The light fastness of fabrics dyed with nano zinc oxide as mordant are grade 1–2, while that of unmordant fabrics is only grade 1. The wash fastness of fabrics dyed with ZnO nanomaterials as mordant were mostly grade 1–2, among which the washing color fastness of sample 9 was grade 2, while the wash color fastness of fabric dyed with ordinary ZnO mordants was only grade 1.

The fastness (wash and light) of silk fabrics dyed with tea polyphenols under the action of different mordants are shown in (Table 3). The results showed that the light fastness of the fabrics dyed with nano zinc oxide as mordant could reach grade 3, while that of the traditional metal mordant was grade 2–3. The wash fastness of dyed fabrics with nano zinc oxide as mordant were above grade 3–4, among which the wash fastness of dyed fabrics with samples ^#^1, ^#^2, ^#^10 and ^#^12 as mordant could reach grade 4–5, the wash fastness of dyed fabrics with samples ^#^3, ^#^13, ^#^14 and ^#^16 as mordant were grade 4, while the color fastness of dyed samples with ordinary zinc oxide as mordant was grade 3.

## 4. Conclusions

In summary, ZnO nanoparticles were prepared by a simple hydrothermal method. We may draw a conclusion that the surface area increases their effect and the biocompatibility of ZnO nanoparticles as mordant was noteworthy. They can improve the dyeing properties and fastness (wash and light) of hematoxylin and tea polyphenols on silk fabric to a certain extent. In addition, the color of dyed fabrics with ZnO nanoparticles as mordant could be maintained very well compared with the metal mordants. Therefore, this method can be an effective way to enhance the dyeing fastness of natural dyes. However, the K/S of the silk fabrics dyed with hematoxylin and tea polyphenols under the action of different morphology nano-zinc oxide mordants showed no significant improvement compared with ordinary ZnO mordant.

## Figures and Tables

**Table 1:** Different reaction conditions of ZnO nanoparticles.

Sample	Temperature/℃	Reaction Time/h	Material Ratios (Zn^2+^:OH^−^/(mol/mol))
^#^1	100	6	2:1
^#^2	100	6	1:1
^#^3	100	6	1:2
^#^4	100	6	1:4
^#^5	130	6	2:1
^#^6	130	6	1:1
^#^7	130	6	1:2
^#^8	130	6	1:4
^#^9	100	12	2:1
^#^10	100	12	1:1
^#^11	100	12	1:2
^#^12	100	12	1:4
^#^13	130	12	2:1
^#^14	130	12	1:1
^#^15	130	12	1:2
^#^16	130	12	1:4

**Table 2:** Color fastness of silk fabrics dyed with hematoxylin under the action of different mordants.

Substrate	Mordant	Light Fastness	Wash Fastness (Color Change)
Silk	-	1	1–2
	ZnO	1–2	1
	^#^1	1–2	1–2
	^#^2	1–2	1–2
	^#^3	1–2	1–2
	^#^4	1–2	1–2
	^#^5	1–2	1–2
	^#^6	1–2	1–2
	^#^7	1–2	1–2
	^#^8	1–2	1
	^#^9	1–2	2
	^#^10	1–2	1–2
	^#^11	1–2	1
	^#^12	1–2	1–2
	^#^13	1–2	1–2
	^#^14	1–2	1–2
	^#^15	1–2	1
	^#^16	1–2	1

**Table 3:** Color fastness of silk fabrics dyed with tea polyphenols under the action of different mordants.

Substrate	Mordant	Light Fastness	Wash Fastness (Color Change)
Silk	-	2–3	3–4
	ZnO	2–3	3
	^#^1	3	4–5
	^#^2	3	4–5
	^#^3	3	4
	^#^4	3	3
	^#^5	3	3–4
	^#^6	3	3–4
	^#^7	3	3–4
	^#^8	3	3–4
	^#^9	3	3
	^#^10	3	4–5
	^#^11	3	3–4
	^#^12	3	4–5
	^#^13	3	4
	^#^14	3	4
	^#^15	3	3–4
	^#^16	3	4

## Data Availability

The data are included in the main text.
